# Developing a Bayesian workshop for full-time staff statisticians

**DOI:** 10.1017/cts.2024.558

**Published:** 2024-06-04

**Authors:** Shokoufeh Khalatbari, Veera Baladandayuthapani, Niko Kaciroti, Eli Samuels, Jane Bugden, Cathie Spino

**Affiliations:** 1 The Michigan Institute for Clinical and Health Research, University of Michigan, Ann Arbor, MI, USA; 2 Department of Biostatistics, University of Michigan, Ann Arbor, MI, USA

**Keywords:** Applied biostatistical sciences network, Bayesian methods, course evaluation, customized training, Clinical and Translational Science Award (CTSA), Michigan Institute for Clinical and Health Research (MICHR), R programming, translational research, translational science, workforce development

## Abstract

**Introduction:**

There are two main schools of thought about statistical inference: frequentist and Bayesian. The frequentist approach relies solely on available data for predictions, while the Bayesian approach incorporates both data and prior knowledge about the event of interest. Bayesian methods were developed hundreds of years ago; however, they were rarely used due to computational challenges and conflicts between the two schools of thought. Recent advances in computational capabilities and a shift toward leveraging prior knowledge for inferences have led to increased use of Bayesian methods.

**Methods:**

Many biostatisticians with expertise in frequentist approaches lack the skills to apply Bayesian techniques. To address this gap, four faculty experts in Bayesian modeling at the University of Michigan developed a practical, customized workshop series. The training, tailored to accommodate the schedules of full-time staff, focused on immersive, project-based learning rather than traditional lecture-based methods. Surveys were conducted to assess the impact of the program.

**Results:**

All 20 participants completed the program and when surveyed reported an increased understanding of Bayesian theory and greater confidence in using these techniques. Capstone projects demonstrated participants’ ability to apply Bayesian methodology. The workshop not only enhanced the participants’ skills but also positioned them to readily apply Bayesian techniques in their work.

**Conclusions:**

Accommodating the schedules of full-time biostatistical staff enabled full participation. The immersive project-based learning approach resulted in building skills and increasing confidence among staff statisticians who were unfamiliar with Bayesian methods and their practical applications.

## Introduction

There are two opposing schools of statistical inference – frequentist and Bayesian – that differ in how probability of an event is used [1]. In the frequentist approach, statisticians use only the data on hand to make predictions. In the Bayesian approach, statisticians use data as well as prior knowledge about the event of interest to make inferences and draw conclusions. While these differences may seem minor, in practice they are vastly different statistical approaches for analyzing data [2]. Relying solely on p-values derived in the frequentist framework for interpreting data and treating them as binary indicators of significance has come under scrutiny in recent years due to their limitations and potential for misinterpretation [3–6]. Additionally, with a rise in interest in prediction, Bayesian methods offer advantages both in modeling and analyses of biomedical data. Examples include incorporation of prior or expert knowledge in biostatistical analyses, probabilistic interpretation of results which is directly relevant to clinical decision-making, development of complex models including hierarchical models to account for various levels of data and variability, exact analyses of small sample data (e.g. for rare disease studies), and finally accommodating the sequential nature of data sampling and analyses (e.g. adaptive clinical trials) [7,8]. Thus, Bayesian methods can provide researchers and statisticians with enhanced tools for making informed decisions and predictions.

Statisticians will increasingly need knowledge and expertise in both statistical approaches, which they may use independently, or by selecting elements of each, for a variety of clinical and translational study designs and analyses. With clinical trials becoming more complex in design and an increased interest in analyzing big data, statisticians who have expertise in both frequentist and Bayesian frameworks will be well positioned to support such research and to select optimal statistical approaches [9].

Many biostatisticians, epidemiologists, and quantitative researchers are only experts in frequentist approaches, because Bayesian coursework was not available during their schooling, and practical training and mentorship opportunities in Bayesian methodology are severely lacking. This lack of training in Bayesian methods could partially be due to the conflict between the two methods in the statistical community in the 80’s, but Bayesian methods have gained more acceptance in recent times [10].

As previously published, the Michigan Institute for Clinical and Health Research (MICHR) recognized that biostatistical collaborators at large diverse academic health centers like University of Michigan (U-M) are necessarily scattered across academic departments as well as the physical campus [11]. This model can isolate applied statisticians, analysts, and many epidemiologists from each other, which may complicate career development and job satisfaction, and inhibit access to optimal biostatistical support for researchers [12,13]. In the era of modern, complex translational research, it is imperative to elevate biostatistical expertise by offering innovative and accessible training [14,15].

Funding for the establishment of the Applied Biostatistical Sciences (ABS) network at U-M was provided by MICHR in 2018. By 2022 the ABS network grew to include over 300 faculty and staff biostatisticians. A survey conducted on this network revealed that many participants had a strong interest in receiving training in Bayesian methods. Other popular topics of interest included: mixed modeling and sample size, and power calculations. The partnership formed between MICHR and statistical collaborators was uniquely positioned to develop, offer, and evaluate this training by working to elevate the expertise of U-M biostatisticians to support evolving research priorities and study designs that are increasingly complex and big data-focused [12]. We engaged U-M biostatistics faculty with Bayesian modeling expertise and teaching experience to participate in the design and implementation of a training course focused on Bayesian analysis methods. To increase the number of U-M biostatisticians who are able to apply Bayesian modeling, it was determined that customized in-person training coupled with application to real-time projects, mentorship, and personalized feedback opportunities should be developed. Leveraging face-to-face interactions, the in-person format facilitated enhanced networking and peer mentorship among participants. Furthermore, it fostered relationship-building with instructors. The workshop was offered pre-COVID, a time when online learning was not widely utilized. Also, since U-M is an integrated campus, it was logistically feasible to offer a completely in-person workshop.

The hands-on training was innovative in its approach, tailored to accommodate the busy schedules of full-time biostatistics staff members at U-M, while also fostering mentoring and networking opportunities. Unlike traditional trainings that are commonly lecture-based, the training was designed to provide an immersive experience where statisticians tackled a defined project of their choosing throughout the course of the training. In this manner, participants gained a deep understanding of the theory and were poised to readily apply the methodology they learned in practice. Following training, we utilized a peer-mentorship model to provide participants channels to ask each other questions and share their knowledge of best practices as they applied Bayesian methods to existing and new projects. The faculty instructors also served as consultants for participants for 6 months to support their knowledge transfer and the implementation of these new skills.

## Background

In 2018, MICHR established the ABS network for a campus-wide community of staff and faculty statisticians, epidemiologists, data scientists, data analysts, and researchers, with the intention of supporting both researchers and biostatisticians, while promoting high-quality clinical and translational science and research [11]. In addition to providing networking opportunities, quarterly technical and collaborative skills trainings were developed and disseminated through the network to elevate the statistical expertise and knowledge among statisticians and researchers. In response to members’ interest, the ABS network leadership team partnered with biostatistical faculty with the objective to provide a workshop series to introduce statisticians to Bayesian principles and analytical tools that could be applied to solve research problems. The ABS network provided an optimal home to deliver the Bayesian training as it aligned with its mission of elevating statistical expertise and creating strong connections and partnerships across its member base.

Four professors from the U-M Department of Biostatistics with expertise in applying Bayesian methods in their collaborative work and experience in teaching were contacted for their interest in designing and implementing this workshop series. All agreed to participate. Three subsequent planning meetings were held to discuss the objectives for the series: length, number, and duration of the sessions, topics to be covered, the materials to be used, pre-course assignments, and the course homework and final project. Since most ABS network statisticians use SAS for programing and Bayesian methods are more commonly done using R programming, the decision was made to provide an introduction to R at the start of the workshop.

This series of lectures and labs was intended for biostatisticians with little to no prior experience with Bayesian analyses. To ensure that the training would provide maximum benefit to the students, it was deemed necessary to have all students at a similar technical level. Prerequisites were instituted for the course that included having a master’s or doctoral degree in biostatistics, statistics, or related fields, and prior analysis experience with frequentist approaches. Students were also required to have their own equipment and a laptop with R Software. We also required written approval from the participant’s direct supervisor or manager before registering for the course. The number of attendees was limited to 25 in order for instructors to provide in-depth instruction and more personalized mentorship. The length of the training was structured to ensure the syllabus for the course could be completed. See Table 1 for the course schedule from August 2019 to September 2020.

**Table 1.** Bayesian course schedule

Syllabus	Month							
	Aug	Sep	Oct	Nov	Dec	Jan	Feb	Mar–Sep
Pre-course reading assigned	x							
Introduction to R, R Lab	x							
Introduce prior and posterior distributions, Review conditional probability distribution		x						
Single parameter and multiple parameter models		x						
Gibbs sampler, MCMC*, diagnostics, and divergence			x					
JAGS and R (relevant to Bayesian methods)			x					
Multiple linear regression				x				
Multiple logistic regression				x				
Random effects (mixed models)					x			
Clinic for help/support						x		
Capstone presentations							x	
Consultation with instructors								x

* Markov Chain Monte Carlo.

Each session was planned for mid-week (on Wednesdays), every other week, for two hours in the late afternoon (3:30–5:30 pm). Most sessions included a one-hour lecture followed by a one-hour hands-on lab where students practiced the concepts and had available time to ask questions. Sessions were held in person once a month in August and December and twice a month in September, October, and November. A contract was signed with a local commercial video production company to record the lecture portions of the workshop series. Videos were available online to students within 24 hours of completion of the individual course sessions so students could review the course material whenever needed.

All training participants were required to complete a capstone project which could be done individually or as a team. To fulfill this requirement, students would have to complete a project analyzing research data using both frequentist and Bayesian approaches and provide a comparison of the results. The instructors would advise the students to select a dataset from one of their projects that had already been analyzed using frequentist methods (and thus knew well) for the capstone project. Course instructors were available to help students identify a statistical problem and work with the students to develop a plan for data analysis during two help clinics. The analysis could be completed using the student’s preferred software (e.g., *R*, SAS). Students gave a 10-minute presentation of their project on the last day of the course. The presentation included background, objectives, methods (frequentist and Bayesian), and a results section. A PowerPoint template (4–6 slides total) was provided so that each student’s presentation would follow the same format. Because this was a pilot workshop, it was hard to estimate how much time would be needed to complete the project; however, the month of January was allotted as working time. It was expected that students would spend 2–4 hours/week on the analysis and presentation. Clinics for help and support for the capstone projects were held in January with the capstone presentations scheduled for February.

*Statistical Inference* by Casella and Berger was chosen as the reference book for the training. The book was provided electronically to attendees at no cost after registration, along with the solutions manual. As pre-course assignments, the students were sent an email with the specific chapters and sections of the book that the instructors deemed essential. The instructors routinely used R for the workshop.

The learning outcomes and core competencies of the workshop included:

- Understanding basic theory for Bayesian statistics
- Use of sound Bayesian approaches for data analysis
- Interpreting the Bayesian data analysis results to address the relevant scientific questions
- Implementing Bayesian computational algorithms using R or SAS
- Performing model checking and model diagnostics
- Understanding differences between frequentist and Bayesian approaches

Because this course was more intensive than other ABS network training opportunities and there were a limited number of student openings for the course, there was concern that students might sign up but later drop out. To incentivize completion, there was an initial course fee of $500 which would be waived upon successful completion of the course. Also, an attendance policy was instituted where students were allowed to miss only one of the eight mandatory lecture/lab sessions offered from September through December. The R Workshop in August (2019) was optional provided that the student was proficient in R programming. The January (2020) help clinics were also optional. It was recommended for students to attend both February (2020) sessions.

## Methods

Survey methods were used to inform the development and evaluate the quality and impact of this novel training in Bayesian approaches. A survey of all current ABS network members was conducted in 2018 to assess their interest in receiving different kinds of biostatistical and professional development support. After the course was designed, it was promoted and prospective students were invited to register. Participant surveys were administered before the start of the training, following each training in the series, and one year following the conclusion of the course. Eleven surveys were administered in total during the workshop series: one pre-workshop (in 2019, before the workshops started), seven post sessions (after each mandatory class), one post-series (in 2020, after the series ended), and one-year post-series (in 2021). Following best practice, regular reports of the results of these surveys and participant metrics were reviewed by faculty and staff leading the implementation of the training for the purpose of formative evaluation [16].

The evaluation of the short-, intermediate-, and long-term outcomes of the program involved assessments of the participants’ training experience, learning, use of Bayesian methods in their capstone project, and their application of Bayesian approaches in their subsequent scientific work [17]. Participants’ consistent participation throughout the duration of the course, and their successful completion of the capstone project and presentation were also key performance metrics that were tracked during the program. Logic models were used by MICHR faculty and staff to distinguish the outcomes of this novel Bayesian training program from those of the ABS network considered overall [18]. Qualtrics [19] was used for conducting all survey instruments.

## Results

Twenty-two ABS network members registered for the workshop series. One student dropped the course halfway through the sessions due to personal issues and one did not show up for any of the training sessions. The capstone projects could be done individually or as a team. Nine participants had individual capstone projects and 11 formed into four groups. All successfully completed their capstone projects and delivered their presentations in-person over a three-hour period. Copies of the presentations were distributed and assigned among the instructors for summative reviews. Emails were then sent to each student with a full review of their project. The pre-program surveys were sent to all 21 registered participants who began the program, of which 16 responded (76% response rate). The post-program survey was sent to the 20 participants who remained in the program, of which 16 responded (80% response rate). The response rates for the seven topic sessions ranged from 50% to 83% and the attendance rates ranged from 80% to 100%, as shown in Table 2.

**Table 2.** Topic session attendance and survey response rates

Topic/Session Number & Title	Registered	Attended	Surveys Completed	Survey Response Rate as % of Attendees
Introduction to R, R lab	18	18	14	78%
Session 1: Prior, Posterior Distribution, Conditional Probability	21	21	17	81%
Session 2: Single & Multiple Parameters Models	21	21	16	76%
Session 3: Gibbs Sampler, MCMC*, Diagnostics, Divergence	21	20	10	50%
Session 4: JAGS and R (relevant to Bayesian Methods)	20	17	10	59%
Session 5: Multiple Linear Regression	20	16	10	63%
Session 6: Multiple Logistic Regression	20	18	15	83%
Session 7: Random Effects	20	18	15	83%

* Markov Chain Monte Carlo.

The results of the post-program survey suggested that participants’ training experience was quite positive. The location and timing of the topic training sessions worked well for a large majority of participants (82% and 88%, respectively). Responding participants indicated that they would recommend the Bayesian workshop to their colleagues. Analyses of respondents’ open-ended survey responses about their training experience indicated it was challenging but feasible to complete, enriched by group discussions, and complimented by their capstone projects.

**Figure 1.**
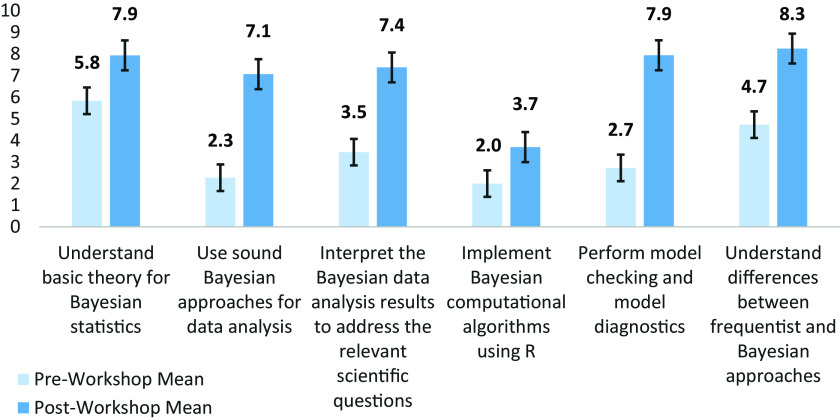
Participants growth in knowledge of and ability in Bayesian methods. Confidence questions, average responses (0 = no confidence; 10 = total confidence). How confident are you that you can perform the following tasks today?.

**Figure 2.**
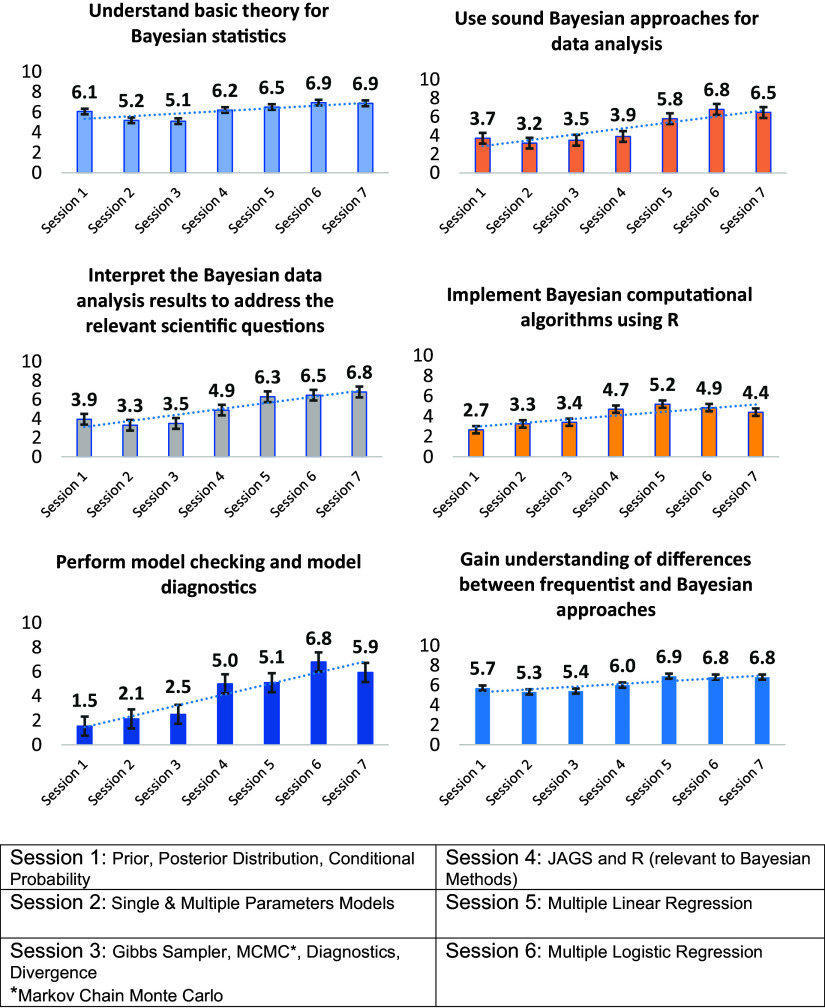
Participants growth in confidence in Bayesian approaches in the training.

Participants’ learning was assessed both by comparing learning outcomes measured by the pre- and post-program surveys and also periodically throughout the program by the topic-session surveys. Comparisons of the learning outcomes assessed in the pre- and post-program surveys indicate participants gained confidence in their use of Bayesian approaches across all outcomes, particularly in their data analysis and model checking and diagnostics, as shown in Figure 1. The assessments of participant learning outcomes measured by the topic sessions’ surveys demonstrate that participants’ confidence fluctuated in magnitude over the duration of the training period, increased at different rates, and reflected different patterns of change over time. As shown in Figure 2, changes in each of the six learning outcomes measured over the duration of the training differ. These differences reflect how the participants discriminated between the course content covered throughout the training. For example, participants’ confidence in their understanding of model checking and diagnostics was the lowest of all those asked at the start of the course but increased substantially in the second half of the course. In contrast, participants’ confidence in their understanding of basic Bayesian theory was the highest of all those assessed at the start of the course and subsequently declined in the first half of the course before increasing in the second half. As discussed further in the Conclusion, these findings lend further support to other empirical research suggesting that clinical and translational researchers’ understanding of research design and theory may not increase simultaneously during research training experiences [20].

The results of the post-program survey also suggest that the participants were successfully able to use Bayesian methods in their capstone project and that many were able to apply Bayesian approaches in their subsequent scientific work. All responding participants reported that their capstone projects had helped them understand and apply Bayesian methods. One year after the workshop series ended, 69% of respondents considered applying Bayesian methods for a study or analysis and 68% reported that they discussed the use of Bayesian methods for a study or analysis with their Principal Investigator (PI) or a colleague. Of those who reported having such a discussion with their PI, several intended to use Bayesian approaches in future studies, two reported their intent to use the Bayesian analysis conducted for the capstone project in a manuscript submission, and one published a manuscript using Bayesian method.

## Conclusion

These survey results and participation metrics uncovered aspects of the training programs that required improvement during the implementation of the program. For example, the results of the topic-session surveys indicated several participants were struggling to learn R programming since they were primarily SAS users. This was brought to the instructor’s attention, and they agreed that SAS had introduced some very good Bayesian procedures that easily fit a lot of the models that were being covered in the course and would be added where possible. Similarly, in response to the participants requesting more time with the instructor for a Group Office Hour/Q&A Session, the instructors added an extra training session. Some respondents also noted that more time was needed to complete readings and assignments, or that the course material was too advanced or basic for this skill level.

The objective of this training program in Bayesian approaches was to provide a novel opportunity for U-M biostatisticians to learn Bayesian modeling. The evaluation results demonstrate that the design and implementation of the training provided participants with a better understanding of Bayesian approaches and skill in the application of Bayesian methods to clinical and translational research studies. These evaluations also revealed opportunities to improve the quality of this training offering, through the proactive use of multiple statistical software packages and dedicated time to both prepare for and hold training sessions.

The results of this evaluation demonstrate the effectiveness of this novel Bayesian method training but must be interpreted in the context of the limitations of this study. The surveys used in the evaluation were administered anonymously to protect the anonymity of the respondents who were being asked to systematically report on their abilities and the quality of the instruction they received from local experts in Bayesian methods. In addition, although the administration of anonymous surveys facilitated high survey response rates, those participants who did not attend each training session could not fill out surveys for the missed sessions. Finally, this evaluation could not control for mitigating factors known to affect clinical and translational researchers’ self-efficacy in their research skills, such as demographic, professional, and organizational variables [21], without potentially compromising the anonymity of the participant data being reviewed by the authors.

Many factors were likely facilitators of the effectiveness of this training program. Perhaps the greatest factor contributing to the success of the program was the ability to identify Bayesian experts within U-M who would teach and be available for the series and for post-workshop mentoring. The following criteria were used in selecting the appropriate instructors: had taught previous Bayesian courses, were active users of the Bayesian methods in clinical and translational research, and had developed Bayesian methods for statistical literature. The presence and service of these experts likely helped to ensure that the participating statisticians felt increasingly confident and continued to consider Bayesian models for their scientific studies. However, the results of this evaluation also suggest that participants’ confidence in their understanding of different topics related to Bayesian methods did not develop consistency or equality over the course of the training. These findings conform to those of other empirical research showing clinical and translational researchers’ understanding of research design and theory do not increase simultaneously during research training experiences [20,22]. However, these results do support the general notion that the particular subjects being studied can affect the ways learners shape their understanding of new topics over time [23].

The administrative and financial support provided by MICHR also contributed to the opportunity to obtain leadership approval and buy-in from departments and academic units across U-M. Costs included modest compensation to the instructors and staff effort. This support was also instrumental to the efficient management of the logistics for the workshop series including planning for the training timing, locations, equipment, supplies, and communications. Clear and consistent communication about the course workload, goals, and expectations also helped the participating statisticians benefit from and complete the training. Finally, the development of an evaluation plan at the beginning of the course design and implementation process ensured that comprehensive and concise participant surveys could be developed and conducted. This evaluation approach helped the instructors, authors, and project team more quickly determine what aspects of the training were working effectively for the participants and which were not.

The design and implementation process used for this novel training in Bayesian approaches can be easily replicated by other clinical and translational research centers within and outside of the Clinical and Translational Science Award Consortium. This process may be applicable to a wide variety of immersive training targeted for full-time professionals and adult learners in the extramural research workforce. Bayesian training could be potentially extended to a broader non-technical clinical /translational investigator audience by focusing on the concepts and their application to specific case studies of interest. We expect that this approach could prove beneficial for clinical/translational investigators, especially those with some quantitative background, as they would collaborate with biostatisticians to perform these analyses. Understanding the advantages and disadvantages of using Bayesian methods framework in comparison to the frequentist approaches would facilitate the collaborative effort between statisticians and the investigators. As an example, understanding the differences in interpretation from the classical frequentist hypothesis testing framework with p-values and confidence intervals vs. credible intervals and posterior distributions of estimates from Bayesian approaches would be critical for a broader audience. Finally, using a workshop format is better suited for a diverse audience of biostatisticians and clinical investigators. Targeting both biostatisticians and clinical investigators would result in better implementation of Bayesian methods, specifically when eliciting and incorporating relevant prior distributions in the analysis. Future research should evaluate the effects of training in Bayesian methods using larger groups of biostatisticians and include principal investigators and other clinical research professionals for the purposes of comparison. Participants’ training experience and scientific productivity should be tracked to evaluate the long-term impact of this training.

Although we reached the targeted participants limit, there are several factors potentially affecting the staff attendance at the workshop series. These include the duration of the workshop, the time commitment, and the $500 course fee imposed on participants who miss multiple classes or fail to complete the Capstone project. Some practical considerations to ensure successful implementation of such training include offering training during work hours, shortening the duration of the workshop series or offering more flexible scheduling options, requiring manager’s support and approval, periodic surveying of students, adaptability to make timely modifications to the training to meet the needs of the students, and ensuring adequate mentoring is part of the training.

Furthermore, with the post-COVID shift to holding sessions and meetings virtually, offering virtual or hybrid sessions (lectures virtually and labs in-person) could be assessed for the impact on the enrollment rate. Online didactic lectures from the workshop series would be relatively easy to accomplish and could extend beyond U-M to accommodate the current hybrid work culture. However, there could be several challenges associated with conducting the lab sessions online; technical issues; lack of immediate feedback; limited interaction and collaboration; and difficulty in demonstrating the more complex concepts. Another teaching modality is having flipped courses, i.e. participants watch the lectures beforehand and in-person/online sessions could be focused on problem-solving or application of methods to data examples. Regardless of the format, the instructors believe that within the timeframe of this workshop series, there would be insufficient time to cover other aspects such as Bayesian approaches to study design, survival analyses, missing data, and hierarchical modeling. Future workshop series could be developed to cover these topics. Another consideration is to develop an introductory level Bayesian workshop tailored for the broader academic medical community to promote the adaptation of these methods through collaborative efforts between statisticians and clinical investigators.

We believe that careful organizing and planning of all aspects of the training from design, scope, staffing resources, budget, timeline, evaluation process, and communications was essential to the program’s success. The U-M collaborative biostatistics faculty experts who were willing to help design and teach the series proved to be the single greatest factor in promoting the development of ABS network statisticians who devoted their time to learn how to use Bayesian approaches for their work. In fact, there is an interest in developing a second workshop series on the design of Bayesian clinical trials and studies to complement the Bayesian analysis-focused topics from this workshop series.

## References

[1] Bland JM , Altman DG. Bayesians and frequentists. BMJ. 1998;317(7166):1151–1160. doi: 10.1136/bmj.317.7166.1151. 9784463 PMC1114120

[2] Fornacon-Wood I , Mistry H , Johnson-Hart C , Faivre-Finn C , O’Connor JPB , Price GJ. Understanding the differences between Bayesian and frequentist statistics. Int J Radiat Oncol Biol Phys. 2022;112(5):1076–1082. doi: 10.1016/j.ijrobp.2021.12.011. 35286881

[3] Andrade C. The P value and statistical significance: misunderstandings, explanations, challenges, and alternatives. Indian J Psychol Med. 2019;41(3):210–215. doi: 10.4103/IJPSYM.IJPSYM_193_19. 31142921 PMC6532382

[4] Nahm FS. What the P values really tell us. Korean J Pain. 2017;30(4):241–242. doi: 10.3344/kjp.2017.30.4.241. 29123617 PMC5665734

[5] Wasserstein RL , Lazar NA. The ASA statement on p-values: context, process, and purpose. Am Stat. 2016;70(2):129–133. doi: 10.1080/00031305.2016.1154108.

[6] Ruberg SJ. Détente: a practical understanding of P values and bayesian posterior probabilities. Clin Pharmacol Ther. 2021;109(6):1489–1498. doi: 10.1002/cpt.2004. 32748400 PMC8246739

[7] US Food and Drug Administration. Guidance for the Use of Bayesian Statistics in Medical Device Clinical Trials. Rockville, MD: Guidance for Industry and FDA Staff, FDA-2006-D-0191, 2010.

[8] Van de Schoot R , Depaoli S , King R , et al. Bayesian statistics and modelling. Nat Rev Methods Primers. 2021;1(1):1. doi: 10.1038/s43586-020-00001-2.

[9] Allenby GM , Bradlow ET , George EI , Liechty J , McCulloch RE. Perspectives on Bayesian methods and big data. Customer Needs Solutions. 2014;1(3):169–175. doi: 10.1007/s40547-014-0017-9.

[10] Berry DA. Bayesian statistics and the efficiency and ethics of clinical trials. Stat Sci. 2004;19(1):175–187.

[11] Khalatbari S , Jazdzyk D , Capsouras J , Downey B , Samuels E , Spino C. Developing an applied biostatistical sciences (ABS) network. J Clin Transl Sci. 2020;5(1):e15. doi: 10.1017/cts.2020.506. 33948241 PMC8057411

[12] Perkins SM , Bacchetti P , Davey CS , et al. Best practices for biostatistical consultation and collaboration in academic health centers. Am Stat. 2016;70(2):187–194. doi: 10.1080/00031305.2015.1077727. 27777443 PMC5074551

[13] Welty LJ , Carter RE , Finkelstein DM , et al. Strategies for developing biostatistics resources in an academic health center. Acad Med. 2013;88(4):454–460. doi: 10.1097/ACM.0b013e31828578ed. 23425984 PMC3610776

[14] Califf RM , Ginsburg GS. Organizational improvements to enhance modern clinical epidemiology. JAMA. 2008;300(19):2300–2302. doi: 10.1001/jama.2008.638. 19017917

[15] Zelen M. The training of biostatistical scientists. Stat Med. 2003;22(21):3427–3430. doi: 10.1002/sim.1642. 14566925

[16] Goldfarb S , Morrison G. Continuous curricular feedback: a formative evaluation approach to curricular improvement. Acad Med. 2014;89(2):264–269. doi: 10.1097/ACM.0000000000000103. 24362392

[17] Kirkpatrick D, Kirpatrick JD. Evaluating Training Programs: The Four Levels (3rd Edition). San Francisco, CA: Berrett-Koehler Publishers, 2006.

[18] McLaughlin JA , Jordan GB. Using logic models, Handbook of Practical Program Evaluation, 2015:62–87.

[19] Qualtrics. Copyright © 2020 Qualtrics. Provo, UT, USA, First Release: 2005.

[20] Dumeny L , Dyson KA , Fantone JC , et al. Training methods that improve MD-PhD student self-efficacy for clinical research skills. J Clin Transl Res. 2019;3(6):316–324. doi: 10.1017/cts.2019.419. PMC6885998 31827905

[21] Livinƫi R , Gunnesch-Luca G , Iliescu D. Research self-efficacy: a meta-analysis. Educ Psychol. 2021;56(3):215–242. doi: 10.1080/00461520.2021.1886103.

[22] Black ML , Curran MC , Golshan S , et al. Summer research training for medical students: impact on research self-efficacy. Clin Transl Sci. 2013;6(6):487–489. doi: 10.1111/cts.12062. 24330695 PMC3868994

[23] Neumann AJF. Staking a claim on learning: what we should know about learning in higher education and why. Rev High Educ. 2014;37(2):249–267.

